# Body Representations in Children with Cerebral Palsy

**DOI:** 10.3390/brainsci10080490

**Published:** 2020-07-28

**Authors:** Antonella Di Vita, Maria Cristina Cinelli, Simona Raimo, Maddalena Boccia, Stefano Buratin, Paola Gentili, Maria Teresa Inzitari, Teresa Iona, Marco Iosa, Daniela Morelli, Francesco Ruggeri, Giuseppina Russo, Cecilia Guariglia, Liana Palermo

**Affiliations:** 1 Department of Psychology, Sapienza University of Rome, via dei Marsi 78, 00185 Rome, Italy; mcricinelli@gmail.com (M.C.C.); maddalena.boccia@uniroma1.it (M.B.); cecilia.guariglia@uniroma1.it (C.G.); 2 IRCCS Santa Lucia Foundation, 00179 Rome, Italy; simonaraimo.sun@gmail.com (S.R.); p.gentili@hsantalucia.it (P.G.); m.iosa@hsantalucia.it (M.I.); d.morelli@hsantalucia.it (D.M.); francesco.ruggerif@gmail.com (F.R.); liana.palermo@unicz.it (L.P.); 3 Department of Medical and Surgical Sciences, Magna Graecia University of Catanzaro, 88100 Catanzaro, Italy; inzitari@unicz.it (M.T.I.); iona@unicz.it (T.I.); 4 OENGINEERING, 36100 Vicenza, Italy; stefano.buratin@gmail.com; 5 ASP di Catanzaro, Unità Operativa Complessa di Neuropsichiatria Infantile, 88100 Catanzaro, Italy; giuseppina.russo@asp.cz.it

**Keywords:** body representation, body schema, body structural description, body image, cerebral palsy

## Abstract

We constantly process top-down and bottom-up inputs concerning our own body that interact to form body representations (BR). Even if some evidence showed BR deficits in children with cerebral palsy, a systematic study that evaluates different kinds of BR in these children, taking into account the possible presence of a general deficit affecting non-body mental representations, is currently lacking. Here we aimed at investigating BR (i.e., Body Semantics, Body Structural Representation and Body Schema) in children with cerebral palsy (CP) taking into account performance in tasks involving body stimuli and performance in tasks involving non-body stimuli. Thirty-three CP (age range: 5–12 years) were compared with a group of 103 typically-developing children (TDC), matched for age and sex. 63.64% of children with CP showed a very poor performance in body representation processing. Present data also show alterations in different body representations in CP in specific developmental stages. In particular, CP and TDC performances did not differ between 5 to 7 years old, whereas CP between 8 and 12 years old showed deficits in the Body Structural Representation and Body Schema but not in Body Semantics. These findings revealed the importance of taking into account the overall development of cognitive domains when investigating specific stimuli processing in children who do not present a typical development and were discussed in terms of their clinical implications.

## 1. Introduction

The body mental representation is a complex process, determined by the interaction of information coming from many different stimuli inside and outside the body [1,2]. A discrete amount of data, including neuropsychological studies on patients with brain damage (e.g., Reference [3]), neuroimaging studies on healthy individuals (e.g., Reference [1]) and developmental studies on healthy children (e.g., References [4,5]), suggests that there is no a single representation of the body in the cortex. In particular, referring to the so-called triadic taxonomy [3,6] we have to distinguish between the Body Schema, which arise from sensory-motor information and it is fundamental to action’s planning and execution; the Body Structural Representation, a topographic map of the different body parts, and the Body Semantics, essentially a conceptual and linguistic representation of the body. These different body representations are supported by a wide network of brain areas (for a metanalysis see Reference [1]) and it is well known that they can be compromised in adult patients with acquired brain damage [3,7].

Although individuals with cerebral palsy (CP) presents body representation alterations [8] that resemble those found in adults with acquired brain injury and although some studies support the idea that, in these individuals, functional deficits in daily activities cannot be explained only in terms of motor difficulties [9], modest attention has been paid to the investigation of body representation deficits in this population.

The majority of the data came from the second reading of works that have investigated motor imagery deficits and that can provide information also about Body Schema deficits in CP. The Body Schema, indeed, can be evaluated using motor imagery tasks such as hands/feet laterality judgments. These motor imagery tasks can be performed by mentally simulating the physical movement and thus, as the actual movements, they involve the use of the Body Schema [3,9]. Mutsaarts and coworkers [10] investigated motor imagery in 19 adolescents with left and right hemiparetic CP using pictures of hands showed in different orientations. Results highlighted that the participants with right hemiparetic CP (left brain damage) did not show the classical linear increase in the reaction times (RTs) as a function of the increasing angle of rotation that was instead shown by the healthy controls and the left hemiparetic CP. This pattern of results would suggest a more severe Body Schema deficit in individuals with right hemiparetic CP (left brain damage). Similarly, Craje and coworkers [11] investigated motor imagery in ten adolescents affected by right hemiparetic CP and controls, analyzing differences in RTs between medial and lateral rotations of hands showed in palm and back view, under the assumption that medial directions (towards the mid-sagittal plane) are easier than rotations in the lateral direction. The authors did not find the medial-lateral rotation effect and concluded that patients with right hemiparetic CP present compromised motor imagery, a finding that suggests a compromised Body Schema. Steenbergen and colleagues [12] instead tested 11 controls and 22 patients with left and right hemiparesis with a hand mental rotation task. The participants with left and right congenital hemiparesis showed the classical linear RT increase but their overall RTs were significantly slower than controls.

Overall, these previous data on motor imagery in CP suggest the presence of Body Schema deficit in this population but are inconclusive on whether these deficits are independent of a more general cognitive deficit involving the imagery domain. Indeed, in all these studies, only hand laterality judgments tasks were used (i.e., no tasks probing the mental rotation of a non-body stimulus like objects were used); thus it is difficult to evaluate the independent contributions of a more general mental imagery deficit to performance.

To the best of our knowledge, only two studies (i.e., References [13,14]) have investigated body representations different from the Body Schema in CP patients. In a recent study, Nuara and coworkers [13] found a Body Structural Representation deficit in ten children with unilateral CP showing that patients, differently from controls, draw themselves more asymmetrically than classmates and hemiparetic peers. The asymmetry index was calculated as the differences between the upper limbs’ length. Furthermore, the children with CP did not show difficulties in left-right discriminations and body-part pointing and naming tasks, a result that is consistent with spared Body Semantics.

Fontes and colleagues [14], who have carried out the only study focused on all body representations (i.e., Body Schema, Body Structural Representation and Body Semantics), found deficit at all levels of the body processing in children with CP, regardless of the laterality of motor, therefore brain, damage. However, these two studies, similarly to the studies on motor imagery in CP, did not use tasks involving non-body stimuli and thus did not take into account the role of deficits in other cognitive domains (i.e., linguistic, attentional, mental imagery) that could explain CP performance. For example, impaired performance in a task assessing the semantic association between body parts and clothes could be due to a more general semantic deficit rather than to a category-specific deficit (i.e., Body Semantics deficit). Indeed, according to Bonato and colleagues [15], the comparison between experimental and control groups does not protect per se against the risk of erroneously attributing the impaired performance to the pathology. It is worth noting that this aspect is particularly relevant in the case of category-specific deficits, such as those involving body-related stimuli. Among the strategies recommended by Bonato and colleagues [15] to prevent this risk, there is the introduction of a “control” task superimposable to the experimental one in terms of stimuli presentation, response modality and so forth.

In sum, although the few studies mentioned above converge in highlighting the presence of body representation deficits in children/adolescents with CP, to the best of our knowledge, no study investigated these representations taking into account the role of a possible deficitperformance in other cognitive domains. Raimo and colleagues [4] investigated this issue in the development of body representations in healthy children from 7 to 10 years old, finding that Body Semantics was completely developed in typically-developing children between 7 and 8 years old. By the age of 9–10 years, children reach an adult-like pattern also in the Body Structural Representation, while the Body Schema was still not completely developed in this range of age.

The aim of the present study was, therefore, to assess body representations (i.e., Body Semantics, Body Structural Representation and the Body Schema) in children with CP comparing performance in tasks involving body stimuli with performance in similar tasks involving non-body stimuli (from now on “control tasks”).

## 2. Materials & Methods

### 2.1. Participants

All children with a diagnosis of CP admitted to the outpatient rehabilitation service of the I.R.C.C.S. Santa Lucia Foundation in Rome and to the Child Neuropsychiatric Service of ASP of Catanzaro from March to October 2018 were suitable to be included in the study. Patients were sequentially sampled from the services mentioned above. The team evaluated the possibility for each child to be enrolled in the study, taking into account all clinical characteristics that could have prevented the administration of the experimental tasks. In particular, the inclusion criteria encompassed: the ability to understand simple verbal commands, the ability to use a touch screen monitor (i.e., to press on a stimulus appearing on the monitor and to press in a specific spatial position), the presence of CP diagnosticated according to the criteria used by the Surveillance of Cerebral Palsy in Europe network [16] and the absence of uncontrolled epilepsy.

The sample included thirty-three CP participants (age range: 5–12 years; mean age = 7.69 years, SD = 2.21 years; 21 males). The sample size is similar to the average size of other studies in the field [10,11,12,13,14]. See Table 1 for demographic and clinical details. A group of 103 typically-developing children (TDC), similar in age (mean = 8.04 years; SD = 1.28 years; Manney-Whitney U = 1404.00; *p* = 0.128) and sex (49 boys; 54 girls; χ^2^ = 2.582; *p* = 0.108) with the CP group, was enrolled. The control sample overlaps with that of a previous study of our research group [4] for children between 7 and 10 years.

The study was designed and conducted in accordance with the ethical principles of the Declaration of Helsinki on the human experimentation. It was approved by the Ethics Committee of “Santa Lucia Foundation” in Rome (Prot. CE/PROG.576, 21 October 2016) and by the Calabria Region Ethical Committee, Catanzaro, Italy (Prot. n. 311, 21 December 2017). All parents of participants signed written informed consent after the procedures had been fully explained to them and children expressed oral consent.

### 2.2. Body Representations Assessment

Body representations (i.e., Body Semantics, Body Structural Representation and Body Schema) were evaluated by means of experimental tasks previously used by our research group in a study on the development of body representations in typically developing children [4]. Here we used the same apparatus, tasks and procedures described in Raimo and colleagues [4], to which reference is made for further details.

**Figure 1:**
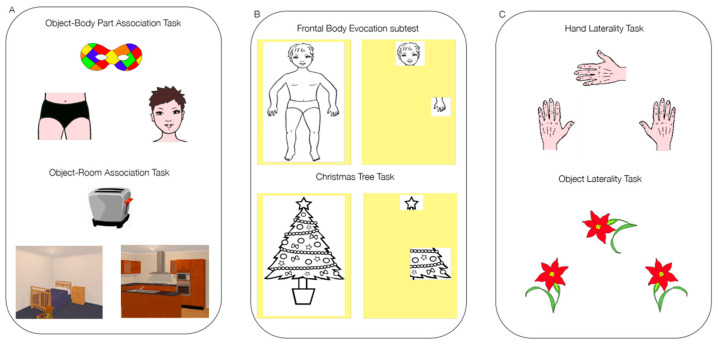
Examples of items for the tasks probing body (top of panel **A**–**C**) and non-body processing (bottom part of panel **A**–**C**). (**A**) Example of the items used to assess Body Semantics and of the corresponding control task, (**B**) Body Structural Representation and of the corresponding control task, (**C**) Body Schema and of the corresponding control task.

Body Semantics was assessed with the Object-Body Part Association Task (adapted from Reference [9]). In this task, the participant had to match an object with the corresponding body part, choosing between two alternative pictures and by pressing on the selected picture on the touch-screen (see Figure 1A). The number of corrected responses (maximum score = 20) was recorded.

The Body Structural Representation was assessed with the computerized version of the Frontal Body Evocation subtest (FBE) of the Body Representation test [19]. This task has also been previously used both in patients with brain damage [2,7,20] and amputees [2,21] to assess this body representation. After observing the drawing of a child for 10 s, an image was shown where only the child’s head was presented as a reference. The participant was asked to re-locate nine body parts of this child by dragging, once at a time, each body part in the right point on the touch-screen (see Figure 1B). The FBE score was expressed in terms of millimeters of deviation from the exact location of the body parts.

The Body Schema was assessed with the Hand Laterality Task (HL-adapted and simplified from Reference [22]; see also Reference [9]). In this task, the participant was asked to judge the laterality of a target hand (20 items; 10 right and 10 left) which could be presented at varying degrees of angular rotation (0, 45, 90, 270, 315). To answer the participant had to press on the drawing of a left or right hand showed on the touchscreen under the target hand (see Figure 1C). The number of corrected responses (maximum score = 20) was recorded.

As suggested in Bonato and colleagues ([15]; see the introduction for details), in order to take into account the role of cognitive abilities not specifically related to the body processing that could affect performance in tasks assessing body representation, three control tasks were also used. In particular, following the approach previously utilized in other studies aimed at investigating body-related processes (see for example [7,23,24]), for each task involving body stimuli, a control task with the same presentation and administration procedures/scoring but with non-body (i.e., object) stimuli was used, that is:

(1) The Object-Room Association Task [4], assessing the semantic processing of non-body stimuli. The participant had to match an object with a room, choosing between two options.
(2) The Christmas Tree Task [4], assessing the structural representation processing of non-body related stimuli. The participant had to re-locate nine parts of a Christmas tree on a touch-screen where only the star tree topper was presented as a reference.
(3) The Object Laterality Task [4], assessing mental rotation (laterality judgments) of non-body related stimuli. The participant had to judge the laterality of a flower.

All tasks were presented on a laptop with a touch screen monitor (13.3” display) and the accuracy was recorded. The task presentation order (i.e., body/non-body) was counterbalanced across participants.

All the participants were assessed in a silent room at rehabilitation service for children with CP and at their school for TD group. During the testing, the children were instructed to maintain the same position. The children were seated in front of the laptop that was placed on a desk and were required to respond as soon as possible.

### 2.3. Statistical Analyses

Considering that the body representations develop differently during the childhood [4,25,26], in order to take into account such differences, the TDC and CP samples were subdivided into two age groups: (i) children from 5 to 7 years old (TDC1; *n* = 38; CP1; *n* = 21) and (ii) children from 8 to 11 years old (TDC2; *n* = 65; CP2; *n* = 12). CP1 and CP2 groups were similar for Gross Motor Function Measure (GMFM; Manney-Whitney U = 117.00; *p* = 0.707)

First of all, the percentage of CP participants with an impaired z-score in the body and non-body tasks (i.e., a z-score ≤ 2) was computed. Performances on body representation tasks were converted into z-scores by using the normative data from TDC group. Furthermore, by means of Friedman tests, differences in the prevalence of poor performance in the different body and non-body representations tasks among CP group were evaluated.

Kolmogorov-Smirnov test was performed to verify normal distribution of residuals of data of the control and CP groups; since the residuals of data were not normally distributed, non-parametric analyses were performed. In order to test for any differences between CP and TDC in the body representation tasks, taking into account the role of other skills necessary to perform the body tasks, three separated rank analyses of covariance (Quade’s test) on the accuracy of performance in the Object-Body Part Association Task, the FBE and the Hand Laterality Task, were performed with Group (CP1, CP2, TDC1, TDC2,) as the between-subjects factor and respectively the performance at the Object Laterality Task, the Christmas Tree Task and the Object-Room Association Task (i.e., the control tasks) as covariates. The Quade’s tests were followed, when appropriate, by pairwise Mann-Whitney U tests to analyze significant effects. SPSS (version 25.0, IBM Corporation, Armonk, NY, USA) was used to perform statistical analyses.

## 3. Results

Overall, 63.64% of children with CP showed a very poor performance in body representation processing (i.e., a z-score ≤ 2 in at least one out of the three body representation tasks; for a similar methodology on adults with brain damage see Reference [3]). In particular, 21.2% of CP showed a performance below 2 z-scores on the Object-Body Part Association Task (Body Semantics), 56.3% on the FBE (Body Structural Representation) and 21.2% on the Hand Laterality Task (Body Schema). Friedman test showed that the prevalence of poor performance in the three body representation tasks significantly differed among CP (χ^2^_(2)_ = 16.94; *p* = 0.00). Post-hoc comparisons (Wilcoxon test) showed that the prevalence of poor performance in the FBE (Body Structural Representation) differed from both the prevalence of poor performance in the Object-Body Part Association Task (Body semantics; Z = −3.464, *p* = 0.001) and in the Hand Laterality Task (Body schema; Z = −3.00, *p* = 0.03). Whereas, the prevalence of poor performance between the Body Semantics and Body Schema tasks did not differ (Z = 0.00; *p* = 1.00).

Regarding non-body tasks, 24.24% of CP showed a performance below 2 z-scores on the Object-Room Association Task (control task for Body Semantics), 41.94% on the Christmas Tree Task (control task for the Body Structural Representation) and 48.48% on the Object Laterality Task (control task for Body Schema). Friedman test showed that the prevalence of poor performance in the three non- body tasks significantly differed among CP (χ^2^_(2)_ = 7.125; *p* = 0.028). Post-hoc comparisons (Wilcoxon test) showed that the prevalence of poor performance in the Object-Room Association Task differed from both the prevalence of poor performance in the Christmas Tree Task (Z = −2.111, *p* = 0.035) and in the Object Laterality Task (Z = −2.828, *p* = 0.050). Whereas, the prevalence of poor performance between the Christmas Tree Task and Object Laterality Task did not differ (Z = −0.277; *p* = 0.782).

Concerning the differences between CP1, TDC1, CP2 and TDC2 in the body representation tasks, the rank analyses of covariance (Quade’s test), which was performed to take into account the performance on other cognitive abilities necessary to perform the body tasks (i.e., the performance on the control tasks), showed a significant difference in each body representation task (Object-Body Part Association Task F_(3, 131)_ = 4.944; *p* = 0.003; FBE F_(3, 125)_ = 6.873; *p* = 0.00; Hand Laterality Task F_(3, 129)_ = 3.938; *p* = 0.010). The Mann-Whitney U tests performed to analyze the significant main effect of Group showed no differences in any body representation tasks between TDC and CP for children from 5 to 7 years old (Object-Body Part Association Task U = 283.00; *p* = 0.063; FBE U = 230.00; *p* = 0.085; Hand Laterality Task U = 266.00; *p* = 0.108). Whereas for children between 8 and 12 years old the Mann-Whitney U tests showed that CP performed worse than TDC in the FBE (U = 214.00; *p* = 0.015) and in the Hand Laterality Task (U = 216.00; *p* = 0.016) but not in the Object-Body Part Association Task (U = 301.500; *p* = 0.113).

**Figure 2:**
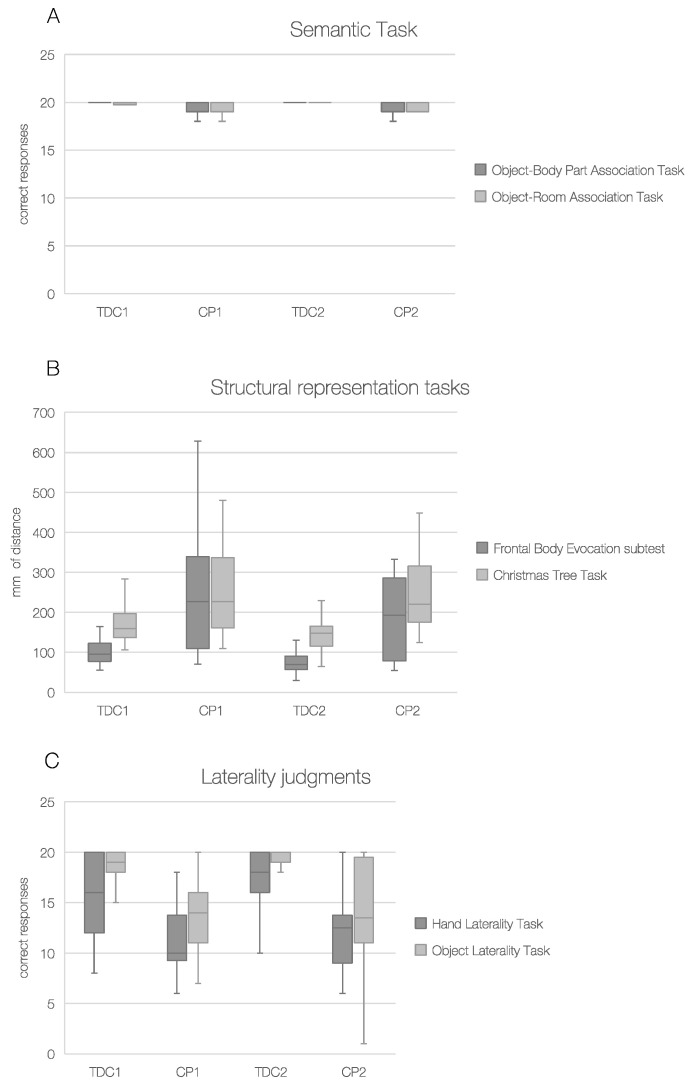
Accuracy in body and non-body tasks. Note: Median number of correct responses for semantic tasks (panel **A**) and laterality judgments (panel **B**) and mm of distance from the correct location for structural representation tasks (panel **C**) are reported. CP1= Children with cerebral palsy from 5 to 7 years old (*n* = 21); TDC1= typically-developing children from 5 to 7 years old (*n* = 38); CP2 = Children with cerebral palsy from 8 to 12 years old (*n* = 12); TDC2 = typically-developing children from 8 to 11 years old (*n* = 65).

The accuracy of performance on the body representation and control tasks for each group are shown in Figure 2.

## 4. Discussion

The processing of information coming from the body and their representation in the cortex is a complex process which, in view of this complexity, cannot be considered a unitary process. In this study we reflect on this complexity investigating different body representations in children with cerebral palsy. Specifically, we aimed to analyze the presence of body representation deficits in this group, but, at variance with previous studies, we were interested in investigating body processing information removing the influence of other cognitive domains. In other words, we were interested in investigating stimulus-specific processes taking into account the role of deficits in other cognitive domains that could affect performance in body representation tasks. With this aim, we evaluated the performance of a group of children with cerebral palsy in tasks with body stimuli taking into account the performance in control tasks, similar in materials and procedures but involving the processing of non-body stimuli (see Reference [15] for such an argument).

Overall, when we considered only the body representation tasks, the percentage of very poor performances was very high in our sample of children with CP (i.e., 63.64% of children with CP showed a very poor performance in at least one out of the three body representation tasks; for a similar methodology see References [3,27,28]). Although this finding should be interpreted with caution since the small sample size of participants with CP, it suggests that the incidence of body representation disorders is high in this population and can be similar to that reported on adult patients with acquired brain damage (e.g., References [3,27,28]). These body representation deficits could be, however, due to more general deficits in skills required to perform the tasks (e.g., visuo-spatial attention, mental imagery, decision making, etc.) regardless of the type of stimuli (i.e., body; non-body). Indeed, the percentage of children with a very poor performance in control tasks and in particular in visuo-spatial (41.94%) and mental rotation (48.48%) tasks, was particularly high. Nevertheless, when we considered the performance in tasks involving body representation processing taking into account the other cognitive processes involved in the body tasks (using non-body stimuli tasks—control tasks), we found that, in the 8–12 age range, children with cerebral palsy performed differently from typically-developing children. In particular, children with cerebral palsy showed a deficit in the Body Structural Representation and in the Body Schema but not in the Body Semantics. The absence of significant differences between children with cerebral palsy and children with typical development in the age group 5–7 can be attributed to the fact that in this age group the Body Structural Representation and the Body Schema are not yet fully developed, as shown by Raimo and colleagues [4] in children with typical development. In other words, impairments may emerge or be more easily detectable when these representations reach an adult-like pattern of development.

The percentage of children with cerebral palsy that showed a very poor performance (i.e., below 2 z-scores) on the task tapping the Body Structural Representation was significantly higher (56.3%) than that of the Body Schema (21.2%) and Body Semantics (21.2%). This result, close together with that of a specific deficit in Body Structural Representation in children with cerebral palsy between 8 and 12 years old, suggests a more pervasive deficit in the Body Structural Representation in cerebral palsy. In other words, the processing of spatial information about the body seems more affected in children with cerebral palsy. This result is in line with that of Nuara and colleagues [13], who evidenced an alteration of spatial relations in the self-portrait of children with cerebral palsy. Present findings also confirm the data collected by Fontes and coworkers [15]. These Authors concluded that patients with cerebral palsy present a Body Structural Representation deficit, finding between-group differences between the group with cerebral palsy and the controls in both a task of finger agnosia and a verbal body part localization task. Nevertheless, it must be noticed that the assessment of the Body Structural Representation in the Fontes and coworkers study [15] also included a visual body-part localization task and a matching body parts by location task whose performance has not been found to differ between the controls and the patients. Besides spatial knowledge of the body, the tasks mentioned above seem to encompass a Body Schema and Body Semantics component that could explain the apparent contrast both with the present and Fontes and colleagues [15] results. In other words, the participants could rely on other body representations to solve the tasks.

Concerning the Body Schema, the present result is consistent with the study by Fontes and coworkers [15] and with the second reading of previous studies that used hand laterality tasks in cerebral palsy [10,11] and that suggested a Body Schema deficit in these patients. However, a possible caveat is that our task involves only the mental motor imagery of the hands and does not evaluate the Body Schema related to other body parts. In order to provide a clearer picture of body representation in cerebral palsy, thus, future studies should evaluate the Body Schema considering also other body parts (e.g., the feet).

As far as the Body Semantics is concerned, to the best of our knowledge, only other two studies have investigated this representation in cerebral palsy. Nuara and colleagues [13] did not investigate the Body Semantics systematically but in the description of the clinical evaluation reported that their sample of children with cerebral palsy was not affected in a body-part naming task (no details were given on tasks and procedure), a result that is consistent with our group analysis that suggests no specific Body Semantics deficit in this population. Similarly, Fontes and coworkers [14] did not point out differences between participants with cerebral palsy and controls in a Body Parts and Object Association task, that is a task superimposable, for the utilized stimuli and procedure, to that used in the present study to assess Body Semantics. In the same study, Fontes and coworkers [14] highlighted, instead, a significantly poor performance of children with cerebral palsy in a body-part naming task in which they were asked to name eighteen isolated body parts. The lack of a control task involving non-body stimuli, however, makes it difficult to understand if this deficit was related to the Body Semantics processing or was due to a more general lexical deficit. Future studies should, thus, investigate Body Semantics in this population using also naming tasks with body and non-body stimuli to thoroughly verify the presence of a deficit affecting this specific body representation.

Some limits should be acknowledged when interpreting our results. Above all our conclusions are drawn on a small sample of children with CP, whereby the present study should be considered as a pilot study for further investigations. The small sample size has also limited the possibility to perform subgroup analyses based on the clinical characteristics of the sample that could affect body representations (i.e., diagnosis). Furthermore, as it has been mentioned above, differently from the Body Semantics and Body Structural Representation tasks, the Body Schema task involved only the mental rotation of hands, this is a limit since this representation was not evaluated for the entire body.

## 5. Conclusions

The presents results suggest that the body representation deficits, mainly involving the Body Structural Representation and Body Schema, can be frequently detected in cerebral palsy. They also highlight the importance of taking into account the overall development of cognitive domains (e.g., language, visuo-spatial and mental imagery skills) beyond the specific stimuli processing in children who do not present a typical development. In addition, considering that the body representation deficits can severely affect functional independence because of their interplay in action execution [29], present results could be relevant to shape new rehabilitation interventions rooted in a clear understanding of the body representation deficits in cerebral palsy and their interaction with functional outcome. As a matter of fact, the intertwining between the Locomotor Body Schema, namely an internal model for controlling movements during walking [30] and the mental representation of walking was found altered in cerebral palsy [31]. Thus, it could be relevant, from both clinical and theoretical perspectives, to investigate the relation between the body representations investigated here and activities of daily life, such as walking, which greatly affect functional outcome.

## Figures and Tables

**Table 1:** Demographic and clinical details of the group with cerebral palsy.

Sex (Proportion)	Mean Age (SD)	Mean Education (SD)	Mean Raven (SD)	QI RANGE (Proportion) [17]	Diagnosis (Proportion)	Mean Gross Motor- GMFCS (SD)	Mean GMFC % (SD)	Mean Abilhand (SD)	Mean Manual Ability Classification System-MACS (SD)
M = 0.64	7.77 (2.24)	1.87 (1.89)	19.74 (5.97)	VIQ58 = 0.03	n.c = 0.09	1.88 (1.16)	80.23 (26.06)	26.77 (8.12)	1.81 (0.95)
F = 0.36				65–75 = 0.09	spastic left = 0.18				
				75–85 = 0.15	spastic right = 0.18				
				85–95 = 0.18	spastic bilateral = 0.42				
				95–105 = 0.24	distonic = 0.06				
				105–115 = 0.30	ataxic n = 0.06				

Note: M = male; F = female; n.c. = not classifiable; VIQ = Verbal Intelligence Quotient measured using the WISC- IV [18].
